# Frequency of Neuropathic Sensory Symptoms Among Patients With Uncontrolled Diabetes Mellitus in Security Forces Hospital, Riyadh, Saudi Arabia

**DOI:** 10.7759/cureus.17528

**Published:** 2021-08-28

**Authors:** Abdulrahman Alamri, Khalid Alharbi, Khaled Hassan, Salem Alhakami, Mohammed Alosaimi, Khalid Rofidi, Ibrahim Ahmed

**Affiliations:** 1 Medicine, Imam Mohammad Ibn Saud Islamic University, Riyadh, SAU; 2 Surgery, Imam Mohammad Ibn Saud Islamic University, Riyadh, SAU; 3 Internal Medicine, Imam Mohammad Ibn Saud Islamic University, Riyadh, SAU; 4 Family Medicine, Imam Mohammad Ibn Saud Islamic University, Riyadh, SAU

**Keywords:** diabetes mellitus, hyperglycemia, diabetes complications, diabetic neuropathies, diabetic peripheral neuropathy (dpn)

## Abstract

Background

Diabetic peripheral neuropathy (DPN) is a chronic sensorimotor length-dependent and symmetrical polyneuropathy. Some peripheral neuropathies have painful presentations, and some are painless. DPN can have a potential impact on the patient's life.

Objectives

This study was conducted in order to investigate the frequency of neuropathic sensory symptoms among patients with uncontrolled diabetes mellitus.

Methods

This is a cross-sectional study conducted in the Security Forces hospital using the Neuropathy Total Symptom Score-6 (NTSS-6) questionnaire. The questionnaire was administered by contacting patients through the phone. Patients with uncontrolled diabetes (HbA1C >9) were included in the study.

Results

This study included 285 participants; 58.9% had type II diabetes and 41.1% had type I diabetes, 156 (54.7%) were females, and 129 (45.3%) were males. Most of the patients (51.1%) were 45-64 years old and the majority were non-smokers (77.9%). Patients with neuropathic pain were 182 (63.9%); 79 (43.4%) of them were males and 103 (56.6%) were females. The prevalence of neuropathic symptoms was much higher in females than in males.

Conclusion

The prevalence of painful DPN is high among patients with long-term uncontrolled diabetes mellitus. Older, unemployed, and low-educated patients are at higher risk of developing painful DPN. Proper glycemic control and lifestyle modifications are essential in preventing the progression of this condition.

## Introduction

Diabetes mellitus (DM) is one of the largest worldwide epidemics in developing and developed countries and one of the most important public health challenges [1]. In 2019, the global prevalence of DM is estimated to be 463 million (9.3%) [2]. An increase in DM's prevalence is expected and estimated to be 578 million (10.2%) and 700 million (10.9%) in 2030 and 2045 consecutively [2]. 

Mortality and morbidity of DM complications are the main contributors to the global burden [1]. Increased prevalence of DM is associated with increased diabetic complications which significantly affect patients' quality of life [3]. Diabetic peripheral neuropathy (DPN) is typically defined as a chronic sensorimotor length-dependent and symmetrical polyneuropathy [4]. DPN have several pathways; some of them have painful presentations, and the others are painless [5]. 

Although DPN is estimated to be the most common diabetic complication, it is still frequently underdiagnosed [6]. In addition, there is a lacuna in estimating the accurate prevalence of DPN which results in great variability in the prevalence reports [7]. The variability has been reported to be from 10-90% which could be explained by the diversity of criteria and how neuropathy is defined [8]. 

According to Wang et al., in Saudi Arabia, the prevalence of DPN was found to be 19.9% [9]. Algeffari reported that 35% of patients with type 2 DM were suffering from painful DPN, poor treatment compliance; additionally, high hemoglobin levels were found to be associated with painful DPN [10]. DPN has a great impact on patients' lives, affecting the psychological and social well-being of the patients, indicating the necessity of a thorough patient assessment [11]. In 2005, Bastyr et al. developed the Neuropathy Total Symptom Score-6 (NTSS-6) questionnaire that provided a valid and reliable evaluation of neuropathic symptoms among patients with DM and DPN [12]. The present study aims to explore the frequency of neuropathic sensory symptoms among patients with uncontrolled DM.

## Materials and methods

Study design

This is a cross-sectional study that was conducted in Security Forces Hospital, Riyadh, Saudi Arabia from December 2019 to February 2020. 

The questionnaire

The questionnaire was in Arabic which initiated with demographic data including age, gender, marital status, smoking status, duration and type of diabetes, and so forth. The Neuropathy Total Symptom Score-6 (NTSS-6) was used to estimate the frequency of neuropathic symptoms. It has six close-ended questions to assess aching pain, allodynia, burning pain, lancinating pain, numbness, and prickling sensation that can be answered by never or occasional, occasional but abnormal, often or almost continuous.

Data gathering

We enrolled all patients with uncontrolled DM (HbA1c > 9) who visited the diabetes clinic at Security Forces Hospital over the study period by registering their telephone numbers. Also, we excluded all patients who had known to have psychiatric illness and were thus incapable of answering questions Then, telephone interviews were held by a physician from the Family Medicine Department. 

Data analysis

The data were entered into Microsoft Excel and analyzed through Statistical Package for the Social Sciences (SPSS) (version 25). Categorical variables including age, gender, type of DM, educational level, etc., were summarized and reported in terms of frequency distribution. A chi-square and fishers' exact tests were used to test the significance of cross-tabulation for neuropathic pain. P-value of <.05 was considered significant.

Statement of ethics

Verbal informed consent was obtained from participants prior to the study, after having a brief explanation about the aims of the study and the contents of this telephone interview. The identity of the patients as well as the raw data, which included personal information, were kept confidential. This consent procedure was reviewed and approved by Al-Imam University Institutional Review Board (IRB), approval number 81-2019.

## Results

A total of 285 participants were included in this current study; 58.9% had type II diabetes and 41.1% had type I diabetes, and 71.9% of patients were on insulin injection treatment. Around 156 (54.7%) of the participants were females, while 129 (45.3%) were males. Most of the patients (51.1%) were 45-64 years old, 77.9% were not smokers, and the majority (79.3%) had a low level of education. Around half the participants (43.9%) were unemployed, 9.8% were housewives and 8.4% were students. Additionally, 182 (63.9%) of patients had neuropathic pain and the remaining 103 (36.1%) had no pain at all.

Among patients experiencing neuropathic pain, 79 (43.4%) were males and 103 (56.6%) were females, most of them (56.0%) were between the ages of 45-64 years followed by those aged 65-96 years (23.6%), (P<.05). The majority (86.3%) of participants with neuropathic pain had secondary or lower education and 46.7% were unemployed, (P<.001). Of the participants with neuropathic pain, 33.5% were type 1. Of the diabetes mellitus patients, 66.5% were type 2 (P<.001) (shown in Table 1).

**Table 1:** Demographic characteristics of the study population (n = 285) All variables were tested to Fisher's exact test.

parameter			All patients	Participants having neuropathic pain(%)	Participants not having neuropathic pain(%)	p-value
Age		0-17	20(7.0%)	10(5.5%)	10(9.7%)	.002
		18-44	60(21.1%)	27(14.8%)	33(32.0%)	
		45-64	146(51.2%)	102(56.0%)	44(42.7%)	
		65-96	59(20.7%)	43(23.6%)	16(15.5%)	
Gender		Male	129(45.3%)	79(43.4%)	50(48.5%)	.403
		Female	156(54.7%)	103(56.6%)	53(51.5%)	
Marital status		Married	192(67.4%)	134(73.6%)	58(56.3%)	.000
		Divorced	5(1.8%)	1(0.5%)	4(3.9%)	
		Widower	41(14.4%)	30(16.5%)	11(10.7%)	
		Single	47(16.5%)	17(9.3%)	30(29.1%)	
Educational status		Secondary education or lower	226(79.3%)	157(86.3%)	69(67.0%)	.000
		Diploma	15(5.3%)	5(2.7%)	10(9.7%)	
		Bachelor	44(15.4%)	20(11.0%)	24(23.3%)	
Occupation		Unemployed	125(43.9%)	85(46.7%)	40(38.8%)	.000
		Housewife	28(9.8%)	22(12.1%)	6(5.8%)	
		Retired	27(9.5%)	21(11.5%)	6(5.8%)	
		Student	24(8.4%)	6(3.3%)	18(17.5%)	
		Civil servant	26(9.1%)	21(11.5%)	5(4.9%)	
		Private-sector employee	9(3.2%)	6(3.3%)	3(2.9%)	
		Military personnel	46(16.1%)	21(11.5%)	25(24.3%)	
Smoking status		Not a smoker	222(77.9%)	143(78.6%)	79(76.7%)	.635
		Former smoker	28(9.8%)	19(10.4%)	9(8.7%)	
		Currently smoking	35(12.3%)	20(11.0%)	15(14.6%)	
Doing regular exercise		No	165(57.9%)	110(60.4%)	55(53.4%)	.247
		Yes	120(42.1%)	72(39.6%)	48(46.6%)	
Type of DM		Diabetes mellitus type 1	117(41.1%)	61(33.5%)	56(54.4%)	.001
		Diabetes mellitus type 2	168(58.9%)	121(66.5%)	47(45.6%)	
Duration of having diabetes mellitus		Less than one year	10(3.5%)	3(1.6%)	7(6.8%)	.019
		6-10 years	41(14.4%)	22(12.1%)	19(18.4%)	
		More than 10 years	234 (82.1%)	41(86.3%)	77(74.8%)	
Mode of current treatment		Oral medications	80 (28.1%)	58(31.9%)	22(21.4%)	.058
		Insulin injections	205 (71.9%)	124(68.1%)	81(78.6%)	
All participants	285 (100%)			182(63.9%)	103(36.1%)	

The majority of patients never experience neuropathic pain, while 40.3% of patients experience occasional prickling or tingling feelings (shown in Table 2).

**Table 2:** Prevalence of different neuropathic symptoms and their frequencies among patients with uncontrolled diabetes mellitus

Parameters	Never or occasional number (%)	Occasional but abnormal number (%)	Often number (%)	Almost continuous number (%)
Do you experience a deep, aching, tightness, boring, pulling, or squeezing pain in your feet or legs?	82(45.3%)	37(20.4%)	24(13.3%)	38(21%)
Do you experience unusual sensitivity or tenderness when your feet are touched or are used in activities such as walking?	88(48.4%)	37(20.3%)	15(8.2%)	42(23.1%)
Do you experience burning pain in your feet or legs?	88(48.4%)	38(20.9%)	27(14.8%)	29(15.9%)
Do you experience sharp, stabbing, or shooting pain, electrical shock-like pain, or surges of pain that last seconds to minutes in your feet or legs?	107(58.8%)	36(19.8%)	24(13.2%)	15(8.2%)
Do you experience numbness, lost sensation, or a 'dead feeling' like an anesthetic, without prickling in your feet or legs?	91(50%)	46(25.3%)	24(13.2%)	21(11.5%)
Do you experience a prickling or tingling feeling, with or without an 'asleep' feeling, in your feet or legs?	35(19.3%)	73(40.3%)	35(19.3%)	38(21%)

Table 3 shows the prevalence of different types of neuropathic symptoms with a comparison between type 1 and type 2 diabetes mellitus (DM) patients. Around 18% of type 1 DM patients experienced continuous deep, aching, squeezing pain in comparison to 22.5% of type 2 DM patients. Around 19.7% of type 1 DM experienced continuous abnormal sensations or tenderness during activities such as walking, whereas the prevalence of continuous pattern of this type of pain among type 2 DM is 24.8%. In addition, 11.5% of type 1 DM patients experienced continuous burning pain in their feet or legs compared to 18.2% of type 2 DM patients. Around 8.2 % of type 1 DM experienced continuous sharp, stabbing, electrical shock-like pain with nearly the same prevalence in type 2 DM patients (8.3%). Regarding continuous numbness and lost sensation, the prevalence was nearly the same in type 1 and type 2 DM patients (11.2% and 11.3%, respectively). Additionally, 18% of type 1 DM patients suffer from continuous prickling or tingling feeling in comparison with 22.5% of type 2 DM patients.

**Table 3:** Frequencies and percentages of different neuropathic symptoms across type 1 and type 2 diabetes mellitus. All variables were tested to fisher's exact test

Parameter		Type1 (n=61)		Type2 (n=121)		p
Do you experience a deep, aching, tightness, boring, pulling, or squeezing pain in your feet or legs?	Never or occasional	29	47.5%	53	44.2%	.914
	Occasional but abnormal	13	21.3%	24	20.0%	
	Often	8	13.1%	16	13.3%	
	Almost continuous	11	18.0%	27	22.5%	
Do you experience unusual sensitivity or tenderness when your feet are touched or are used in activities such as walking?	Never or occasional	31	50.8%	57	47.1%	.830
	Occasional but abnormal	12	19.7%	25	20.7%	
	Often	6	9.8%	9	7.4%	
	Almost continuous	12	19.7%	30	24.8%	
Do you experience burning pain in your feet or legs?	Never or occasional	33	54.1%	55	45.5%	.517
	Occasional but abnormal	11	18.0%	27	22.3%	
	Often	10	16.4%	17	14.0%	
	Almost continuous	7	11.5%	22	18.2%	
Do you experience sharp, stabbing, or shooting pain, electrical shock-like pain, or surges of pain that last seconds to minutes in your feet or legs?	Never or occasional	38	62.3%	69	57.0%	.808
	Occasional but abnormal	12	19.7%	24	19.8%	
	Often	6	9.8%	18	14.9%	
	Almost continuous	5	8.2%	10	8.3%	
Do you experience numbness, lost sensation, or a 'dead feeling' like an anesthetic, without prickling in your feet or legs?	Never or occasional	26	42.6%	65	53.7%	.500
	Occasional but abnormal	18	29.5%	28	23.1%	
	Often	10	16.4%	14	11.6%	
	Almost continuous	7	11.5%	14	11.6%	
Do you experience a prickling or tingling feeling, with or without an 'asleep' feeling, in your feet or legs?	Never or occasional	10	16.4%	25	20.8%	.380
	Occasional but abnormal	24	39.3%	49	40.8%	
	Often	16	26.2%	19	15.8%	
	Almost continuous	11	18.0%	27	22.5%	

Table 4 demonstrates the prevalence of different types of neuropathic pain symptoms among males and females. Around 1.3% of males experience continuous deep, aching, squeezing pain, whereas 22.5% of females experience this type of neuropathic pain. Regarding the continuous feeling of unusual sensation or tenderness on touching the feet or during activities such as walking, the prevalence was high in females (40.8%) but absent in our male patients. Around 2.5% of males experience continuous burning pain in their feet or legs compared to 26.2% of female patients. None of the male participants experience continuous sharp, stabbing, electrical shock-like pain, whereas 14.6% of females do. Regarding continuous numbness and lost sensation, the prevalence was low in males (1.3%) and high in females (19.4%). In addition, 8.9% of males experience continuous prickling or tingling feelings in comparison to 30.4% of females. The prevalence of neuropathic symptoms was much higher in females than in males. The difference between the prevalence of all these symptoms in the males and females was statistically significant (P<.001).

**Table 4:** Frequencies and percentages of different neuropathic symptoms across both genders All variables were tested to chi-square test

Parameter		Males (n=79)		Female (n=103)		p
Do you experience a deep, aching, tightness, boring, pulling, or squeezing pain in your feet or legs?	Never or occasional	57	72.2%	25	24.5%	.000
	Occasional but abnormal	14	17.7%	23	22.5%	
	Often	7	8.9%	17	16.7%	
	Almost continuous	1	1.3%	37	36.3%	
Do you experience unusual sensitivity or tenderness when your feet are touched or are used in activities such as walking?	Never or occasional	63	79.7%	25	24.3%	.000
	Occasional but abnormal	13	16.5%	24	23.3%	
	Often	3	3.8%	12	11.7%	
	Almost continuous	0	0.0%	42	40.8%	
Do you experience burning pain in your feet or legs?	Never or occasional	56	70.9%	32	31.1%	.000
	Occasional but abnormal	12	15.2%	26	25.2%	
	Often	9	11.4%	18	17.5%	
	Almost continuous	2	2.5%	27	26.2%	
Do you experience sharp, stabbing, or shooting pain, electrical shock-like pain, or surges of pain that last seconds to minutes in your feet or legs?	Never or occasional	62	78.5%	45	43.7%	.000
	Occasional but abnormal	10	12.7%	26	25.2%	
	Often	7	8.9%	17	16.5%	
	Almost continuous	0	0.0%	15	14.6%	
Do you experience numbness, lost sensation, or a 'dead feeling' like an anesthetic, without prickling in your feet or legs?	Never or occasional	53	67.1%	38	36.9%	.000
	Occasional but abnormal	15	19.0%	31	30.1%	
	Often	10	12.7%	14	13.6%	
	Almost continuous	1	1.3%	20	19.4%	
Do you experience a prickling or tingling feeling, with or without an 'asleep' feeling, in your feet or legs?	Never or occasional	12	15.2%	23	22.5%	.001
	Occasional but abnormal	39	49.4%	34	33.3%	
	Often	21	26.6%	14	13.7%	
	Almost continuous	7	8.9%	31	30.4%	

Table 5 illustrates the prevalence of different types of neuropathic pain symptoms across various smokers and non-smokers. Around 26.1% of non-smokers experienced continuous deep, aching, squeezing pain; 5.3% of former smokers experienced this type of neuropathic pain, whereas none of the current smokers report such pain. Regarding continuous unusual sensation or tenderness on touching the feet or during activities such as walking, the prevalence was 29.4% among non-smokers, whereas these symptoms were absent in former and current smokers. Additionally, 19.6% of non-smokers experienced continuous burning pain in their feet or legs compared to 5.3% of the former smokers, while current smokers experienced none. None of the former and current smokers experienced continuous sharp, stabbing, electrical shock-like pain, whereas 10.5% of the non-smokers reported doing so. In a similar manner, none of the former and current smokers experienced continuous numbness and lost sensation, whereas 14.7% of the non-smokers did. Finally, 24.6% of the non-smokers experienced continuous prickling or tingling feelings compared to 5.3% of the former smokers and 10% of the current smokers. The difference between the prevalence of all these symptoms, except continuous prickling or tingling feeling, in the non-smokers, former smokers, and current smokers was statistically significant (P<.001).

**Table 5:** Frequencies and percentages of different neuropathic symptoms according to smoking status. All variables were tested to chi-square test.

Parameter		Not a smoker (n=143)		Former smoker (n=19)		Current smoker (n=20)	
Do you experience a deep, aching, tightness, boring, pulling, or squeezing pain in your feet or legs?	Never or occasional	57	40.1%	13	68.4%	12	60.0%
	Occasional but abnormal	28	19.7%	3	15.8%	6	30.0%
	Often	20	14.1%	2	10.5%	2	10.0%
	Almost continuous	37	26.1%	1	5.3%	0	0.0%
Do you experience unusual sensitivity or tenderness when your feet are touched or are used in activities such as walking?	Never or occasional	56	39.2%	16	84.2%	16	80.0%
	Occasional but abnormal	31	21.7%	3	15.8%	3	15.0%
	Often	14	9.8%	0	0.0%	1	5.0%
	Almost continuous	42	29.4%	0	0.0%	0	0.0%
Do you experience burning pain in your feet or legs?	Never or occasional	61	42.7%	15	78.9%	12	60.0%
	Occasional but abnormal	32	22.4%	2	10.5%	4	20.0%
	Often	22	15.4%	1	5.3%	4	20.0%
	Almost continuous	28	19.6%	1	5.3%	0	0.0%
Do you experience sharp, stabbing, or shooting pain, electrical shock-like pain, or surges of pain that last seconds to minutes in your feet or legs?	Never or occasional	75	52.4%	13	68.4%	19	95.0%
	Occasional but abnormal	33	23.1%	3	15.8%	0	0.0%
	Often	20	14.0%	3	15.8%	1	5.0%
	Almost continuous	15	10.5%	0	0.0%	0	0.0%
Do you experience numbness, lost sensation, or a 'dead feeling' like an anesthetic, without prickling in your feet or legs?	Never or occasional	62	43.4%	17	89.5%	12	60.0%
	Occasional but abnormal	42	29.4%	1	5.3%	3	15.0%
	Often	18	12.6%	1	5.3%	5	25.0%
	Almost continuous	21	14.7%	0	0.0%	0	0.0%
Do you experience a prickling or tingling feeling, with or without an 'asleep' feeling, in your feet or legs?	Never or occasional	29	20.4%	4	21.1%	2	10.0%
	Occasional but abnormal	53	37.3%	9	47.4%	11	55.0%
	Often	25	17.6%	5	26.3%	5	25.0%
	Almost continuous	35	24.6%	1	5.3%	2	10.0%

## Discussion

Diabetes mellitus (DM) is a chronic metabolic disorder characterized by impaired glucose tolerance and disturbance of carbohydrate, protein, and fat metabolism, resulting from a lack of insulin or dysregulated insulin signaling [13]. Complications of DM are categorized into two main categories: macrovascular and microvascular complications of DM. Diabetic neuropathy is one of the microvascular complications and is the most common complication of DM and the most common neuropathy, resulting from long-term poor glycemic control [14]. Diabetic neuropathy affects predominantly the peripheries (hands and lower limbs) in a bilateral and symmetrical distribution; however, cranial nerves may be involved [15]. Consequently, the most common presentation is peripheral symmetrical polyneuropathy. Diabetic peripheral neuropathy (DPN) tends to occur after the age of 50 and mainly in patients with type 2 DM as shown in our cases [16]. As reported by Wang et al., the prevalence of DPN in Saudi Arabia is 19.9% [9].

The symptoms of DPN include numbness, tingling, and pain. The clinical findings of DPN include the following: loss of temperature and sensation for pinprick, loss of vibration, and loss of proprioception. Our study demonstrates the frequency of each symptom of DPN in different groups of patients with uncontrolled diabetes mellitus (HbA1c > 9) who visited the diabetes clinic over the study period. Painful DPN is a distressing symptom to patients suffering from diabetic neuropathy. It has two forms: an acute form that resolves within a year or a chronic form that lasts for years. Studies such as that conducted by Ziegler et al. reported that painful DPN is more prevalent in patients with type 2 diabetes than in patients with type 1, and in females than in males as also seen in our cases [16]. Moreover, it has been shown in our cases that painful DPN is more common in the following groups of patients: (1) patients with secondary or low education; (2) patients with long-term uncontrolled DM; (3) unemployed patients; and (4) patients with aged 50 years or more. In the present study, 64% of patients with DM are suffering from painful DPN, whereas Algeffari reported in their study that the prevalence of painful DPN among diabetic patients was 35% [10]. Therefore, these results indicate a high prevalence of painful DPN among diabetic patients.

Neuropathic pain should be clinically distinguished from non-neuropathic pain. Therefore, careful history, examination, diabetes screening, and evaluation of nerve function are essential. There are different types of neuropathic pain that patients may experience, such as deep-aching, tight, pulling, squeezing, burning, stabbing, shooting, and shock-like pain. Assessing the type and severity of pain is done mainly through specifically developed questionnaires that allow patients to describe their experience by neuropathy sensory symptom scale, the Neuropathy Total Symptom Score-6 (NTSS-6), which evaluates individual neuropathy sensory symptoms in patients with diabetes mellitus (DM) and diabetic peripheral neuropathy (DPN) such as (ie, numbness and/or insensitivity; prickling and/or tingling sensation; burning sensation; aching pain and/or tightness; sharp, shooting, lancinating pain; and allodynia and/or hyperalgesia). Questions delivered in a standardized and easy way to the patients in order to get precise and accurate answers would be valuable while monitoring the response to therapy. As painful DPN is associated with discomfort, anxiety, and depression, it has an impact on patients' quality of life and resembles a great burden to the healthcare system [17]. In a similar manner to pain assessment, quality-of-life assessment is made through questionnaires and assessing tools such as the Nottingham Health Profile or the Medical Outcomes Study Short Form [18].

There is no specific treatment for DPN. However, proper glycemic control and lifestyle modifications, such as exercise and appropriate diet, are required to prevent or delay the progression of DPN [19]. In addition, therapies such as α-lipoic acid, opioids, botulinum toxin A, Mexidol, or reflexology could be used [20]. In cases with painful DPN, serotonin, noradrenaline reuptake inhibitors, tricyclic antidepressants, and anticonvulsants are used for relieving pain and discomfort [21].

## Conclusions

The prevalence of painful DPN is high among diabetic patients and is more common among patients with secondary or low education, patients with long-term uncontrolled DM, unemployed patients, and patients aged 50 years or more. Painful DPN negatively affects patients' quality of life and could represent a great burden to the healthcare system. Proper glycemic control and lifestyle modifications are essential in the prevention of the progression of the disease.
